# Toward a holistic understanding of wellbeing: a psychometric evaluation of the 7DHW model in a Portuguese sample

**DOI:** 10.3389/fpsyg.2026.1855916

**Published:** 2026-06-30

**Authors:** Inês Santos Silva, Frank Schifferdecker-Hoch, Luísa Soares

**Affiliations:** 1 2256c.health Unipessoal Lda, Lisbon, Portugal; 2 Departamento de Psicologia, Faculdade de Artes e Humanidades, Universidade da Madeira, Funchal, Portugal

**Keywords:** health knowledge, holistic wellbeing, mental and physical health, psychometrics, questionnaire validation

## Abstract

**Introduction:**

The growing recognition of wellbeing as a multidimensional construct has highlighted the need for integrative and culturally adapted assessment tools. This study aimed to validate the Portuguese version of the Holistic Scale of Body and Mental Health and Wellbeing, based on the 7 Dimensions of Holistic Wellbeing (7DHW) model.

**Methods:**

A cross-sectional study was conducted with 408 Portuguese adults. The psychometric properties of the instrument were evaluated through exploratory and confirmatory factor analyses, internal consistency assessment using Cronbach's α and McDonald's ω, and known-groups validity analyses based on gender and age comparisons.

**Results:**

Exploratory and confirmatory factor analyses supported the multidimensional structure of the questionnaire, with all seven dimensions demonstrating theoretically coherent factorial solutions. Internal consistency was good to excellent for Self-Esteem (α = 0.869), Social Relationships (α = 0.883), Environment (α = 0.829), and Sense of Future (α = 0.898), while Meaningful Work showed acceptable reliability (α = 0.767). In contrast, Body Image (α = 0.097) and Health Knowledge (α = 0.535) demonstrated lower internal consistency, indicating the need for further refinement. Known-groups analyses revealed significant gender differences in Environmental Attitudes and Health Behaviors, with women reporting higher scores, and significant age-related differences in Self-Esteem, Social Relationships, Environment, and Sense of Future.

**Conclusions:**

The findings provide preliminary support for the construct validity and reliability of the Portuguese version of the 7DHW questionnaire. The instrument shows strong potential as a comprehensive measure of holistic wellbeing across psychological, social, environmental, occupational, and behavioral domains. However, further refinement of the Body Image and Health Knowledge dimensions is recommended before broader application.

## 1 Introduction

Over recent decades, the understanding of health and wellbeing has shifted considerably: what was once dominated by a narrow biomedical view has increasingly given way to a broader, holistic perspective that recognizes the interplay of physical, psychological, social, and environmental factors (Engel, 1977; Kickbusch, 1989; McLeroy et al., 1988). Consistent with this, the World Health Organization (WHO) defines health as

“*a state of complete physical, mental, and social wellbeing and not merely the absence of disease*.” (World Health Organization, 1946).

This definition has stimulated interest in multidimensional wellbeing models in research and practice (Ryff, 1995; Seligman, 2011; Keyes, 2002; Rocha and Soares, 2026; Sobral and Soares, 2026).

In the field of psychology and health sciences, numerous models have been proposed to capture the complexity of wellbeing, recognizing that multiple interconnected domains contribute to a person's quality of life (Dodge et al., 2012; Ryff and Singer, 1998; Diener et al., 1999). This more integrative approach is particularly relevant in contexts where physical and mental health, social relationships, and environmental conditions converge, for example, among individuals recovering from chronic or complex conditions where emotional and social recovery may be as important as physical rehabilitation (Santos Silva et al., 2025, 2024; Rodrigues et al., 2025; Silva et al., 2025; Soares, 2026).

In Portugal, recent national reports indicate persistent wellbeing and mental health challenges despite improvements in material living conditions. The Portuguese Wellbeing Index suggests that quality-of-life indicators have stagnated in recent years (Instituto Nacional de Estatística, 2023), while the 2023 Portugal Health Profile reports high levels of anxiety and depression alongside ongoing barriers to mental health support access (European Commission et al., 2023). Mental and behavioral disorders also represent a substantial proportion of the national disease burden (Universidade de Coimbra, 2023).

These findings signal that in Portugal, wellbeing is not simply a matter of physical health but is deeply entangled with psychological, social, and environmental factors. Although several wellbeing instruments have been developed, including Ryff's Psychological Wellbeing Scales, Keyes' flourishing framework, WEMWBS, and WHO-5, many primarily focus on psychological or subjective wellbeing dimensions and may not fully integrate environmental, occupational, and health-literacy components within a single framework (Ryff, 1995; Keyes, 1998; Huppert and So, 2013; Hicks et al., 2013). Recent literature highlights the growing need for validated and culturally adaptable multidimensional wellbeing instruments applicable across clinical, occupational, and public health settings (Diener and Chan, 2011; Bai and Lazenby, 2015). However, tools that coherently integrate both physical and mental health dimensions within a single framework remain relatively scarce (Brown and Applegate, 2012; Kaveh et al., 2016).

In response to this gap, the 7 Dimensions of Holistic Wellbeing (7DHW) model was developed, drawing on the WHO's holistic health principles (dos Santos Silva et al., 2024; Silva et al., 2025). The model proposes seven foundational and interlocking domains: (A) stable self-esteem (Rosenberg, 1965b; Orth et al., 2012), (B) positive body image (Cash and Smolak, 2011; Tiggemann, 2004), (C) supportive social relationships (Cohen and Wills, 1985; Kawachi and Berkman, 2001), (D) an intact and safe environment (Evans, 2003; Frumkin, 2001), (E) meaningful work in healthy conditions (Wrzesniewski et al., 1997; Steger et al., 2012), (F) access to health knowledge and care (Nutbeam, 2000; Kickbusch et al., 2013), and (G) a present and future worth living (Seligman, 2011; Snyder et al., 2002). The model sees these dimensions as mutually influencing and collectively forming a coherent structure for assessing holistic wellbeing.

Based on this framework, the Holistic Scale of Body and Mental Health and Wellbeing (7DHW Questionnaire) was developed and preliminarily tested in a Portuguese sample, demonstrating initial feasibility and test–retest reliability (dos Santos Silva et al., 2024; Silva et al., 2025). The present study extends this work by evaluating the psychometric properties of the Portuguese version using exploratory and confirmatory factor analyses, internal consistency measures, and known-groups validity analyses in a diverse adult sample (Paiva et al., 2014; Orth and Robins, 2014).

The aims of this study are:

- To investigate whether the hypothesized seven-dimension internal structure of the 7DHW Questionnaire is supported in a Portuguese adult population.
- To evaluate the internal consistency and reliability of each sub-scale in the Portuguese version (Cronbach, 1951).
- To provide evidence of construct validity by examining relationships between the wellbeing dimensions and health-related variables within the Portuguese population context.

This study contributes to the development of culturally adapted multidimensional wellbeing assessment tools by evaluating the psychometric properties of the Portuguese version of the 7DHW Questionnaire. The instrument may offer potential applicability across clinical, occupational, educational, and public health contexts in Portugal.

## 2 Background

Across the last few decades, the study of wellbeing has expanded remarkably, becoming a central focus within psychology, medicine, public health, education, and organizational research (Seligman, 2011; Dodge et al., 2012; Breslow, 1999). This shift reflects a broader understanding that human health cannot be reduced to biological processes alone. Instead, wellbeing represents an integrated state encompassing physical vitality, emotional balance, social connectedness, and environmental harmony. In clinical contexts such as psycho-oncology and stroke recovery, this holistic view has gained particular traction, where emotional and social adaptation are now recognized as determinants of long-term rehabilitation and quality of life (Paiva et al., 2023; Soares et al., 2024; Silva et al., 2020; Santos Silva et al., 2025; Rodrigues et al., 2025; Santos Silva et al., 2024).

The WHO described health not as the absence of disease but a state where we combine physical, mental, and social wellbeing (World Health Organization, 1946). This definition remains a cornerstone for contemporary approaches that seek to evaluate wellbeing across interconnected life domains rather than through isolated indicators (Ryff and Singer, 1998).

### 2.1 Defining wellbeing and wellness

Although often used interchangeably, the constructs of wellbeing and wellness highlight different dimensions of the human experience. Wellbeing usually denotes a subjective evaluation of one's life satisfaction, emotional states, and psychological functioning (Dodge et al., 2012; Soares et al., 2026). In contrast, wellness refers to a dynamic, self-directed process of making conscious choices toward a healthy, meaningful life (Helne, 2018). Both perspectives acknowledge that individuals flourish through balance across multiple domains, such as psychological, physical, emotional, social, occupational, and spiritual, and therefore call for integrative frameworks that transcend disciplinary boundaries (Hicks et al., 2013; Brown and Applegate, 2012).

Recent literature also emphasizes the importance of understanding wellbeing historically and contextually, as a lived, evolving construct shaped by cultural, social, and environmental factors (Soares, 2023). Foundational theoretical contributions, such as Ryff's Psychological Wellbeing Model (Ryff, 1995) and Keyes' Flourishing Framework (Keyes, 1998), have highlighted autonomy, personal growth, and positive relations as central pillars of psychological health. Recent reviews also highlight that flourishing is increasingly conceptualized as a multidimensional construct integrating hedonic, eudaimonic, relational, and functional dimensions of wellbeing, although conceptual and measurement inconsistencies remain across instruments and cultural contexts (Rule et al., 2024). Meanwhile, psychometric tools like the Warwick–Edinburgh Mental Wellbeing Scale (WEMWBS) and the WHO-5 Wellbeing Index have advanced population-level measurement. However, they tend to focus primarily on emotional and mental domains (Mental, 2015; Bech, 2012). What many of these approaches lack, however, is the inclusion of environmental, occupational, and future-oriented aspects, dimensions that are essential for a truly holistic assessment (dos Santos Silva et al., 2024; Rosenberg, 1965a).

In Portugal, national health indicators show persistent challenges in mental and emotional wellbeing, especially among younger adults and caregivers (European Commission et al., 2023). These realities reinforce the importance of adopting conceptualisations of wellbeing that transcend psychological metrics and incorporate physical health, environmental security, and access to care, dimensions central to the 7DHW model (dos Santos Silva et al., 2024).

### 2.2 Tools and approaches to measuring wellbeing

Over the past two decades, there has been a proliferation of instruments for assessing Wellbeing across different populations and settings (Hicks et al., 2013; Diener and Chan, 2011). These range from broad psychological wellbeing surveys to context-specific instruments that evaluate workplace engagement (Bakker and Demerouti, 2007), patient-reported outcomes (Velikova et al., 2004), or wellbeing across life stages (Tiggemann, 2004). Despite this diversity, systematic reviews highlight persistent methodological challenges: the lack of multidimensional frameworks, inconsistent psychometric evidence, and limited cross-cultural validation (Nutbeam, 2000; Kickbusch et al., 2013).

Recent literature increasingly supports multidimensional approaches to wellbeing assessment, arguing that unidimensional indicators may fail to capture the complexity and interdependence of psychological, social, environmental, and existential aspects of human flourishing (Schonhardt et al., 2023; VanderWeele and Johnson, 2025).

Despite progress in measurement theory, many existing instruments were developed and validated primarily in English-speaking or Northern European contexts (Huppert and So, 2013; Apfel and Tsouros, 2013). Cultural differences in how individuals interpret wellbeing-related terms, such as “flourishing” or “life satisfaction,” often affect construct equivalence and response validity (Huppert and So, 2013). Cross-cultural adaptation, therefore, requires not only linguistic translation but also conceptual alignment to ensure that items capture culturally meaningful dimensions of Wellbeing. This issue is particularly relevant in Southern European societies, including Portugal, where relational, family, and community factors play a central role in perceptions of health and happiness (Helne, 2018; Bejaković, 2021).

Notable innovations include the Comprehensive Wellbeing Scale (CWBS) (Sham et al., 2021), which integrates intrapersonal and transpersonal dimensions, and the Perceived Wellness Survey (PWS) (Kaveh et al., 2016), used in conjunction with initiatives like NIOSH's Worker Wellbeing programme (Zwetsloot and Leka, 2010). These developments point to a clear scientific demand: reliable, theoretically grounded, and culturally sensitive instruments capable of capturing the multidimensionality of Wellbeing. Increasingly, research also explores the role of digital and social technologies in wellbeing promotion, such as peer-support networks and mobile communication platforms that foster emotional connection and resilience (Santos Silva et al., 2024; Rodrigues et al., 2025).

Another relevant multidimensional wellbeing instrument is the Pemberton Happiness Index (PHI) (Hervás and Vázquez, 2013), which integrates remembered and experienced wellbeing across hedonic, eudaimonic, and social domains and has demonstrated cross-cultural applicability across several countries. While the PHI offers an important contribution to integrative wellbeing assessment, the 7DHW model (dos Santos Silva et al., 2024) differs by explicitly incorporating environmental wellbeing, health knowledge/access, and meaningful work as central dimensions of holistic health.

### 2.3 A holistic framework: from theory to application

While numerous tools have been proposed to measure wellbeing, few truly embody the World Health Organization's holistic conception of health, which situates wellbeing at the intersection of physical, psychological, social, and environmental factors (Antonovsky, 1987; Lindström and Eriksson, 2006; Antonovsky, 1979; Santo et al., 2025). Most instruments tend to isolate these domains rather than capture their interdependence, resulting in fragmented understandings of human flourishing. Contemporary wellbeing frameworks increasingly emphasize environmental sustainability and ecological security as central components of human wellbeing and quality of life (Benczur et al., 2025).

The 7 Dimensions of Holistic Wellbeing (7DHW) model (dos Santos Silva et al., 2024) was conceived to overcome these limitations and to operationalize wellbeing as an integrated, dynamic construct. This model identifies seven mutually reinforcing dimensions: Self-Esteem, Body Image, Social Relationships, Environmental Wellbeing, Meaningful Work, Health Knowledge and Care Access, and Sense of the Future. These dimensions collectively represent the full spectrum of factors contributing to a person's holistic health. It acknowledges that wellbeing emerges from continuous interactions between inner psychological resources and outer living conditions. In this view, self-worth and body acceptance are intertwined with supportive relationships, environmental safety, professional fulfillment, and equitable access to health and knowledge systems (Evans, 2003; Wrzesniewski et al., 1997; Steger et al., 2012).

Although existing instruments such as the WEMWBS (Mental, 2015), WHO-5, Ryff's Psychological Wellbeing Scales (Ryff, 1995), and Keyes' flourishing framework (Keyes, 1998) provide important contributions to wellbeing assessment, most primarily focus on psychological and emotional functioning. In contrast, the 7DHW model (dos Santos Silva et al., 2024) was designed to integrate psychological, environmental, occupational, health-literacy, relational, and future-oriented dimensions within a single holistic framework. This broader integrative perspective may be particularly relevant for contexts where wellbeing is shaped by complex interactions between individual, social, behavioral, and environmental determinants.

From this theoretical foundation, the Holistic Scale of Body and Mental Health and Wellbeing was developed as a psychometric tool designed to measure these dimensions collectively. The instrument was structured to reflect both subjective experiences and objective conditions of wellbeing, enabling the assessment of multidimensional health across diverse populations. Importantly, its validation in the Portuguese context provides an opportunity to examine how cultural, social, and occupational characteristics shape the interconnections among mental, physical, and social wellbeing, which are key to advancing inclusive models of health promotion and public policy.

### 2.4 The Portuguese context: wellbeing, health, and social realities

Portugal presents a particularly relevant context for applying and validating holistic wellbeing tools. Despite notable improvements in healthcare outcomes and life expectancy, reaching 81.7 years in 2022, above the EU average, mental health indicators remain concerning (European Commission et al., 2023). According to the OECD and European Observatory on Health Systems (2023), anxiety and depression rates in Portugal are among the highest in Europe, and access to psychological care remains limited, particularly outside major urban centers (European Commission et al., 2023). Furthermore, mental and behavioral disorders account for roughly 11.8% of the national burden of disease, surpassing oncological diseases (10.4%) (Universidade de Coimbra, 2023; Institute for Health Metrics and Evaluation, 2023).

Among younger populations, the HBSC 2021–2022 survey reported a sharp increase in unhappiness (from 18.3% to 27.7%) and self-harm (from 19.6% to 24.6%) among Portuguese adolescents (Gaspar et al., 2022). Combined with growing economic pressures, an aging population, and the lingering effects of the COVID-19 pandemic, these figures underscore the urgency of developing culturally adapted tools to assess and promote holistic wellbeing in Portugal (European Commission et al., 2023; Bejaković, 2021).

### 2.5 Applications and potential use cases

Given its multidimensional structure, the 7DHW Questionnaire can have broad utility:

- **Organizational wellbeing**: to assess psychosocial safety, job meaning, and engagement, supporting prevention of burnout and improvement of workplace culture (Baras et al., 2018; Zwetsloot and Leka, 2010; Burlakova et al., 2020; Roque and Soares, 2011). Recent workplace wellbeing research also highlights the growing importance of organizational culture, hybrid work environments, and digital wellbeing monitoring in shaping employee wellbeing experiences (Kawakami et al., 2023).
- **Clinical and rehabilitation contexts**: to complement biomedical indicators with psychological, social, and environmental wellbeing dimensions, especially relevant for chronic illness recovery (Lawrence and Kinn, 2012; Brasier et al., 2016; Stanton et al., 2015).
- **Personal development and coaching**: to promote awareness, reflective practices, and identify areas for intervention in self-care and preventive health programmes (Brown and Applegate, 2012; Kawachi and Berkman, 2001; Ryff and Singer, 2006).

### 2.6 Psychometric and cultural validation of the 7DHW in Portugal

Although preliminary studies have supported the internal coherence and theoretical structure of the 7DHW model, a comprehensive validation for the Portuguese population remains a crucial step. Cultural, linguistic, and contextual factors can influence how individuals interpret wellbeing-related items, affecting the reliability and validity of self-report instruments. Therefore, adapting and validating the 7DHW in Portugal is essential to ensure that its seven dimensions retain conceptual equivalence while reflecting national values, linguistic nuances, and the country's specific sociocultural dynamics (Huppert and So, 2013; Kaveh et al., 2016; Orth and Robins, 2014).

Establishing the factorial structure, internal consistency, and construct validity of the instrument will confirm whether the seven-dimensional model accurately captures how wellbeing manifests within Portuguese society. Beyond statistical adequacy, such validation also provides insights into how different aspects of wellbeing, specifically self-esteem, body image, social relations, environment, meaningful work, health knowledge, and sense of the future, interact in this context. Cross-cultural validation is therefore not only a methodological necessity but also an opportunity to examine the intersection of psychological and sociocultural determinants of wellbeing (Baras et al., 2018).

Recent cross-cultural validation studies reinforce the importance of adapting multidimensional wellbeing instruments to local sociocultural contexts, as conceptualizations of wellbeing may vary across populations and cultural settings (Scalas et al., 2023; Sollis et al., 2024).

From a practical perspective, having a validated instrument adapted to the Portuguese context will contribute to evidence-based health promotion and social policy. Portugal's current challenges, ranging from high rates of anxiety and depression to socioeconomic inequality and an aging population, underscore the need for tools capable of assessing wellbeing holistically across individual and community levels (European Commission et al., 2023; Instituto Nacional de Estatística, 2023). By establishing the psychometric robustness of the 7DHW questionnaire, this study supports both scientific advancement in wellbeing measurement and the implementation of culturally grounded interventions in healthcare, education, and organizational settings. The present research aims to strengthen the bridge between theoretical models of holistic wellbeing and their application in real-world Portuguese contexts, advancing not only psychometric scholarship but also the national capacity to monitor and promote integrated forms of health and human prosperity.

## 3 Methods

The present study aimed to evaluate the psychometric properties of the Holistic Scale of Body and Mental Health and Wellbeing within a Portuguese adult population. A cross-sectional design was employed to examine the instrument's internal structure, reliability, and construct validity. All analyses followed established psychometric validation guidelines, including exploratory and confirmatory factor analyses, internal consistency assessments, and group-based differentiation analyses. Statistical procedures were conducted using SPSS (Version 29) and Jamovi, integrating both exploratory and inferential approaches to ensure robustness and reproducibility.

### 3.1 Participants

Participants were recruited from the Portuguese population through online dissemination channels, including institutional mailing lists, social media platforms, direct contact, and professional networks. Inclusion criteria required participants to be adults (≥18 years) and fluent in Portuguese language.

A total of 408 individuals participated in the study. The sample covered a broad age range from 18 to 90 years (M = 43.60, SD = 18.99), ensuring representation across different life stages. The gender distribution was 70.1% female and 29.9% male, reflecting a gender imbalance toward female participants, which should be considered when interpreting group-based analyses.

Regarding health status, 82 participants (20.1%) reported a diagnosed physical condition, most commonly including musculoskeletal disorders, cardiovascular conditions, and chronic pain-related issues. Additionally, 44 individuals (10.8%) reported mental health conditions, such as anxiety, depression, or burnout. Medication use was reported by 160 participants (39.2%), while 171 participants (41.9%) indicated having undergone psychotherapy at some point in their lives.

The sample suggests variability in occupational and educational backgrounds. A total of 328 participants (80.4%) were professionally active, whereas 80 (19.6%) were retired. Educational attainment ranged from basic schooling to advanced academic degrees, with a substantial proportion holding higher education qualifications, including 106 participants with a master's degree, 89 with a licenciatura (bachelor equivalent), and 13 with a doctoral degree. Overall, the sample reflects a heterogeneous adult population, enabling the instrument to be examined across diverse sociodemographic and health-related contexts.

### 3.2 Instrument

The instrument used in this study was the Holistic Scale of Body and Mental Health and Wellbeing, Portuguese version, based on the theoretical framework of the 7 Dimensions of Holistic Wellbeing (7DHW).

For the Portuguese validation, the questionnaire was administered in European Portuguese, following linguistic and cultural adaptation. This process included translation, back-translation, and iterative review to ensure semantic, conceptual, and contextual equivalence with the original version.

The scale assesses seven core dimensions of wellbeing:

1. **Self-esteem:** stable sense of self-worth and emotional resilience;
2. **Body image:** perception and acceptance of one's own body;
3. **Social relationships:** quality and depth of interpersonal connections;
4. **Environment:** perception of environmental safety and ecological awareness;
5. **Meaningful work:** sense of purpose and satisfaction in professional life;
6. **Health knowledge:** awareness, behaviors, and access to healthcare;
7. **Sense of the future:** hope, meaning, and engagement with life.

Items were mapped to dimensions according to the 7DHW model's theoretical structure. Particular attention was given during adaptation to ensure cultural relevance, especially in domains such as healthcare access, work conditions, and environmental perceptions.

Each item was rated on a 7-point Likert scale, ranging from 1 (“Strongly Disagree”/“Never”) to 7 (“Strongly Agree”/“Always”), depending on item formulation. Higher scores indicate higher perceived wellbeing.

To provide a clearer overview of the questionnaire structure, Table 1 summarizes the number of items included in each of the seven wellbeing dimensions, as well as one illustrative example per dimension. This mapping allows readers to understand how each theoretical construct was operationalised in the Holistic Scale of Body and Mental Health and Wellbeing, ensuring transparency in the distribution of items and demonstrating how abstract components of the 7DHW model were translated into concrete, participant-facing questions.

**Table 1:** Overview of the seven wellbeing dimensions, number of items, and example items included in the Holistic Scale of Body and Mental Health and Wellbeing.

Dimensions	Number of items	Examples of items (Portuguese/English)	Answer (Portuguese/English)
A	12	“Sente compaixão por si mesmo quando erra?” (“Do you feel compassionate about yourself when you fail?”)	Likert scale 1 (Nunca/Never) - 7 (Sempre/Always)
B	5	“Sente-se satisfeito e confortável com o seu corpo?” (“Do you feel satisfied and comfortable with your body?”)	Likert scale 1 (Nunca/Never) - 7 (Sempre/Always)
C	8	“Sente que tem autonomia com seus amigos mais próximos?” (“Do you feel you have autonomy with your close friends?”)	Likert scale 1 (Nunca/Never) - 7 (Sempre/Always)
D	8	“A qualidade do ar nas principais cidades cosmopolitas do mundo terá um impacto negativo na saúde.” (“Air quality in major world cosmopolitan cities will negatively impact health.”)	Likert scale 1 (Discordo Totalmente/Strongly Disagree) - 7 (Concordo Totalmente/Strongly Agree)
E	11	“Sente que o seu trabalho tem um propósito significaPvo para você?” (“Do you feel your work has a meaningful purpose for you?”)	Likert Scale 1 (Nunca/Never) - 7 (Sempre/Always)
F	18	“Sente necessidade de fazer exames de sangue uma vez por ano?” (“Do you feel the need to make blood analysis once a year?”)	Likert scale 1 (Nunca/Never) - 7 (Sempre/Always)
G	24	“Já experimentou alguma situa cão intrinsecamente graPficante?” (“Do you ever experience a situation as intrinsically rewarding?”)	Likert scale 1 (Nunca/Never) - 7 (Sempre/Always)

### 3.3 Procedure

Data were collected through an online survey administered via Google Forms, ensuring accessibility, ease of dissemination, and standardized administration across participants. The questionnaire was distributed through multiple digital channels, including social media platforms (e.g., LinkedIn, Facebook, and Instagram), institutional and personal mailing lists, and professional networks. In addition, participants were encouraged to share the survey within their networks, resulting in a snowball sampling effect that broadened outreach to diverse demographic groups.

Participation in the study was entirely voluntary, and no financial or material compensation was provided.

Before accessing the questionnaire, participants were presented with an informed consent form, outlining the study's objectives, procedures, confidentiality measures, and data protection practices. Only individuals who explicitly agreed to participate were allowed to proceed.

No personally identifiable information was collected at any stage of the study. All data were handled in accordance with ethical standards for research involving human participants and complied with the General Data Protection Regulation (GDPR). Ethical approval was obtained from [institution/committee, if applicable].

The average completion time for the questionnaire was approximately 20 to 30 min.

### 3.4 Data analysis

Data analysis followed a structured psychometric validation pipeline:

1. **Descriptive statistics:** Means and standard deviations were computed for all items and dimensions. Distributional properties were examined to identify potential deviations from normality.
2. **Internal consistency:** Reliability was assessed using Cronbach's α, McDonald's ω, and corrected item–total correlations (CITC). Thresholds followed standard recommendations (α≥0.70; ω≥0.70; CITC>0.30).

3. **Construct validity (internal structure):**

- Exploratory Factor Analysis (EFA): Conducted using Principal Axis Factoring with oblique rotation (Promax), following assessment of sampling adequacy via the Kaiser–Meyer–Olkin (KMO) index and Bartlett's test of sphericity.
- Confirmatory Factor Analysis (CFA): Performed to test factorial stability and evaluate the adequacy of the proposed dimensional structure. Models were estimated using robust maximum likelihood (MLR). Model fit was assessed using CFI, TLI, RMSEA (with 90% CI), and χ^2^ statistics.

4. **Differentiation analysis (known-groups validity):** Group comparisons were conducted to examine whether the scale differentiates between demographic groups:

- **Gender:** independent-samples *t*-tests (Welch correction when needed);
- **Age groups:** one-way ANOVAs with *post hoc* Tukey HSD tests. Effect sizes were reported using Cohen's *d* and η^2^.

5. **Assumption testing and robustness checks:** Normality, homoscedasticity, and multicollinearity were evaluated using standard diagnostics (Shapiro–Wilk test, Levene's test, and correlation matrices). When assumptions were violated, robust or non-parametric alternatives were considered.

All statistical analyses were conducted using a significance level of *p* < 0.05 (two-tailed).

## 4 Results

This section presents the results in four stages, aligned with the primary objectives of the validation process. First, exploratory analyses were conducted to examine the distributional characteristics of the data and the behavior of items across dimensions. Second, construct validity was evaluated through factor structure and internal consistency measures. Third, item-level analyses were performed to further assess reliability and conceptual alignment. Finally, group-comparison analyses (e.g., *t*-tests and ANOVAs) were used to examine the instrument's sensitivity to demographic differences, such as age and gender. Together, these steps provide a comprehensive evaluation of the psychometric properties of the Portuguese version of the scale.

### 4.1 Exploratory data analysis

An Exploratory Data Analysis (EDA) was conducted to examine the dataset's preliminary characteristics. This included inspecting descriptive statistics at the dimension level and identifying missing data and potential outliers. The dataset showed no evidence of systematic missingness, with all dimensions fully completed across participants, enabling a complete and reliable analysis.

For analytical clarity, items were grouped into the seven theoretical dimensions of holistic wellbeing:

- Dimension A: Self-Esteem.
- Dimension B: Body Perception.
- Dimension C: Social Relationships.
- Dimension D: Environment.
- Dimension E: Work and Working Conditions.
- Dimension F: Health Knowledge.
- Dimension G: Hope and Meaning.

**Figure 1:**
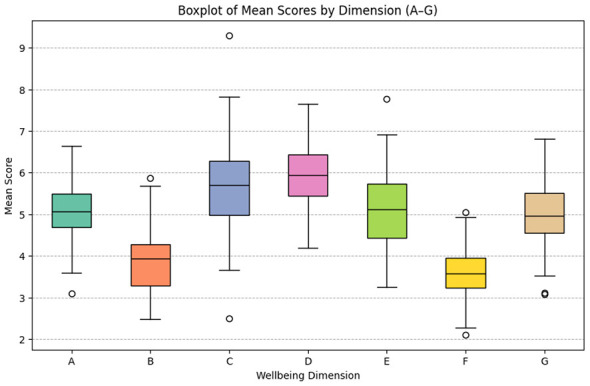
Boxplot of mean scores by Dimension (A–G).

Scores for each dimension were computed as the mean of their respective items. The distribution of these scores is illustrated in Figure 1, which shows a generally balanced spread across dimensions, with moderate variability and no pronounced skewness.

Descriptive statistics revealed the following mean scores (mean ± SD):

- Self-Esteem (A): 5.17 ± 0.79
- Body Perception (B): 3.88 ± 0.73
- Social Relationships (C): 5.60 ± 0.96
- Environment (D): 5.89 ± 0.80
- Work (E): 5.18 ± 0.84
- Health Knowledge (F): 3.99 ± 0.70
- Hope and Meaning (G): 4.59 ± 0.76

Among the seven dimensions, Environment (D) exhibited the highest mean score, suggesting a strong orientation toward ecological awareness and environmental engagement within the Portuguese sample. Social Relationships (C) and Work (E) also presented relatively high scores, indicating positive perceptions of interpersonal connections and occupational wellbeing. In contrast, lower mean values were observed in Health Knowledge (F) and Body Perception (B), which may reflect areas where participants experience greater challenges or limited resources.

To further examine the presence of extreme values, an Interquartile Range (IQR) analysis was conducted for each dimension. Outliers were defined as observations falling below *Q1*−1.5 × *IQR* or above *Q3*+1.5 × *IQR*. The analysis revealed a small number of lower-end outliers across several dimensions, particularly in Environment (D) and Social Relationships (C), indicating that a subset of participants reported notably lower wellbeing in these areas. No substantial clustering of extremely high values was observed.

Despite the presence of these extreme observations, their frequency was limited and did not substantially distort central tendency or dispersion metrics. Consequently, all cases were retained for subsequent analyses in order to preserve ecological validity and ensure that the full spectrum of participant experiences is represented. This decision is particularly relevant in wellbeing research, where variability and outlier cases can provide meaningful insights into vulnerable subgroups. Therefore, all observations were retained for subsequent analyses, preserving the variability inherent to real-world wellbeing experiences.

The boxplot presented in Figure 1 further supports these findings, illustrating relatively consistent distributions across dimensions with moderate interquartile ranges. Slightly greater dispersion is visible in dimensions such as Social Relationships (C) and Work (E), suggesting more heterogeneous experiences in these domains.

Taken together, these results indicate that the dataset demonstrates adequate variability, balanced distributions, and no critical data quality issues. This provides a solid foundation for subsequent analyses of reliability and construct validity.

### 4.2 Construct validity—statistical analysis by dimension

Construct validity was examined by assessing the internal structure and coherence of the instrument's seven theoretically defined dimensions within the Portuguese sample (*N* = 408). Following established psychometric procedures, each dimension was analyzed independently through a combination of exploratory and confirmatory factor analyses. This approach allowed the evaluation of whether the latent structures underlying each dimension were consistent with the theoretical framework of the 7DHW model.

Exploratory Factor Analyses (EFA) were conducted using the minimum residual (MINRES) extraction method with oblimin rotation, allowing for correlated latent factors. Sampling adequacy was assessed using the Kaiser–Meyer–Olkin (KMO) index, and Bartlett's test of sphericity was used to confirm the suitability of the data for factor analysis. Factor retention was guided by eigenvalues greater than one and inspection of scree plots. Confirmatory Factor Analyses (CFA) were subsequently performed to evaluate factorial stability and to test whether each dimension could be adequately represented by a multiple interrelated structure. Model fit was assessed using standard indices (CFI, TLI, and RMSEA) and evaluated against conventional thresholds for acceptable fit.

Furthermore, Cronbach's alpha α and McDonald's ω coefficients were calculated as indicators of internal consistency, with values around or above 0.70 (α≥0.70 and ω≥0.70) considered indicative of acceptable reliability for research purposes. Item–total correlations and inter-item statistics were also examined to assess the homogeneity of each dimension. This multi-step approach ensured that the scale aligned with the theoretical model while demonstrating adequate empirical reliability and validity at the sub-scale level across samples.

Overall, results consistently supported multidimensional structures across all dimensions, reinforcing the theoretical assumption that holistic wellbeing is composed of multiple interrelated yet distinct components rather than single latent constructs.

#### 4.2.1 Dimension A

An exploratory factor analysis (EFA) using the minimum residual (MINRES) extraction method with oblimin rotation was conducted on the 12 items of Dimension A to examine the latent structure underlying self-related psychological competencies in the Portuguese sample (*N* = 408). The Kaiser–Meyer–Olkin (KMO) measure of sampling adequacy was good (KMO = 0.870), and Bartlett's test of sphericity was significant (χ^2^ = 1, 908.44, *df* = 66, *p* < 0.001), indicating that the correlation matrix was appropriate for factor analysis.

Based on eigenvalues greater than one (5.11, 1.23, 1.02) and inspection of the scree plot, an initial three-factor solution was identified, explaining 45.6% of the total variance. This solution grouped items into broader domains reflecting (1) self-perception, confidence, autonomy, and coping, (2) self-awareness and emotional insight, and (3) boundary-setting. Factor loadings were generally substantial, and inter-factor correlations ranged from 0.49 to 0.60, suggesting moderate associations among constructs. However, model fit indices indicated that this solution, while interpretable, did not provide optimal fit (χ^2^ = 160.96, *df* = 33, *p* < 0.001; RMSEA = 0.098; TLI = 0.861), suggesting potential under-extraction of latent dimensions.

To better align with the instrument's theoretical structure, a four-factor solution was also examined. This model accounted for 45.8% of the total variance and yielded a conceptually coherent structure comprising (1) self-awareness and relational skills, (2) boundary-setting, (3) autonomy and coping with adversity, and (4) self-perception and self-acceptance. Factor loadings remained adequate, and inter-factor correlations ranged from 0.42 to 0.67, indicating moderate relationships between constructs while preserving discriminant validity. Nevertheless, the exploratory model fit indices were slightly weaker than desired (χ^2^ = 139.61, *df* = 24, *p* < 0.001; RMSEA = 0.109; TLI = 0.827), reflecting some overlap between dimensions.

A confirmatory factor analysis (CFA) was subsequently conducted to evaluate the stability and adequacy of the four-factor structure identified in the EFA. The correlated four-factor model demonstrated acceptable fit (χ^2^ = 145.26, *df* = 48, *p* < 0.001; CFI = 0.948; TLI = 0.928; RMSEA = 0.071), with standardized factor loadings ranging from 0.40 to 0.88, all statistically significant (*p* < 0.001). These results indicate satisfactory convergent validity and support the adequacy of the proposed multidimensional structure.

In contrast, the unidimensional CFA model demonstrated poor global fit (χ^2^ = 452.28, *df* = 54, *p* < 0.001; CFI = 0.787; TLI = 0.740; RMSEA = 0.135), suggesting that Dimension A is not adequately represented by a single latent factor. Rather, the findings indicate that the construct is internally multidimensional, encompassing multiple interrelated yet distinguishable aspects of self-related functioning. The substantial deterioration in model fit under the unidimensional solution further supports the interpretation that these components cannot be meaningfully reduced to a single homogeneous dimension.

Taken together, the EFA and CFA findings converge in supporting a multidimensional architecture for Dimension A. While the exploratory results indicate some degree of overlap among factors, the confirmatory analysis demonstrates that a four-factor model provides a theoretically meaningful and empirically adequate representation of the data. These findings suggest that Dimension A reflects a set of interrelated but distinct psychological competencies, including self-perception, emotional awareness, relational functioning, autonomy, and resilience. The multidimensional nature of this construct is therefore not a limitation but rather an accurate reflection of the complexity of self-related processes within holistic wellbeing.

Overall, the statistical analysis provides preliminary support for the factorial organization of Dimension A in the Portuguese population and confirms that it is best conceptualized as a four-factor construct with moderate inter-factor correlations, consistent with contemporary multidimensional models of psychological functioning and self-regulation.

#### 4.2.2 Dimension B

An EFA was conducted on the Dimension B items using the MINRES extraction method with oblimin rotation to investigate the latent structure underlying body image, body-related social experiences, and perceived inclusivity. The KMO measure of sampling adequacy was acceptable (KMO = 0.701), and Bartlett's test of sphericity was significant (χ^2^ = 912.37, *df* = 10, *p* < 0.001), confirming that the correlation matrix was appropriate for factor analysis.

Based on eigenvalues greater than one (2.41 and 1.28), a two-factor solution was retained, explaining 52.2% of the total variance (Factor 1: 30.8%; Factor 2: 21.4%). Specifically, the first factor captured body-related social experiences, particularly experiences of body shaming in private and public settings, as well as difficulties in accessing suitable clothing. In contrast, the second factor reflected body satisfaction and perceived inclusivity. Factor loadings were moderate to high (0.58 to 0.93), and item uniqueness values remained within acceptable limits (0.18–0.54), collectively indicating satisfactory representation of the latent structure.

Model fit indices provided additional support for the adequacy of the two-factor solution. Specifically, the RMSEA was 0.000, the TLI was 1.00, and the chi-square statistic was non-significant (χ^2^ = 0.89, *df* = 1, *p* = 0.345), suggesting excellent fit and no evidence of model misspecification. Furthermore, the inter-factor correlation was moderate and negative (*r* = −0.52), indicating that body-related stigma and positive body satisfaction represent related but clearly distinguishable constructs. Taken together, this pattern supports the interpretation of Dimension B as a bidimensional domain rather than a single unified construct.

A subsequent CFA was conducted to evaluate the stability of the two-factor structure identified in the EFA and to assess whether the items could be adequately represented by a unidimensional model. The correlated two-factor CFA model demonstrated good fit (χ^2^ = 6.72, *df* = 4, *p* = 0.151; CFI = 0.992; TLI = 0.978; RMSEA = 0.041), with standardized factor loadings ranging from 0.58 to 0.93, all statistically significant (*p* < 0.001). In contrast, the unidimensional model demonstrated substantially poorer fit (χ^2^ = 48.13, *df* = 5, *p* < 0.001; CFI = 0.903; TLI = 0.806; RMSEA = 0.142), indicating that a single-factor solution does not adequately reproduce the covariance structure of the items.

Taken together, the EFA and CFA findings suggest that Dimension B is better conceptualized as an internally multidimensional construct composed of at least two distinguishable but related facets: body-related social experiences and internal body satisfaction. The moderate association between these factors indicates conceptual relatedness, whereas the negative inter-factor correlation and poor fit of the unidimensional model suggest that these components should not be interpreted as a psychometrically homogeneous dimension.

These findings therefore provide preliminary evidence that body-related wellbeing within the 7DHW framework may involve multiple interrelated sub-domains rather than a single latent construct. Given the low internal consistency observed for the overall dimension, these results should nevertheless be interpreted cautiously and require further investigation in future psychometric studies using independent samples and potential item refinement procedures.

#### 4.2.3 Dimension C

An EFA was conducted for the Dimension C items using the MINRES extraction method with oblimin rotation to assess the latent structure underlying interpersonal connectedness and autonomy in relationships. Sampling adequacy was high, as reflected by a global KMO value of KMO = 0.884, and Bartlett's test of sphericity confirmed the factorability of the correlation matrix (χ^2^ = 3, 612.55, *df* = 28, *p* < 0.001).

Based on eigenvalues greater than one (3.12, 2.18, and 1.41), a three-factor solution was retained, accounting for 73.8% of the total variance (Factor 1: 29.4%, Factor 2: 24.1%, Factor 3: 20.3%). The resulting factors represented three conceptually coherent domains: (1) emotional closeness and relational support, (2) social belonging and connectedness, and (3) autonomy in interpersonal relationships. Items loaded strongly onto their respective factors, with loadings ranging from 0.62 to 0.94 and low uniqueness values (0.10–0.42), indicating strong communalities and robust construct representation.

The factor structure further revealed moderate correlations between the latent dimensions (*r* = 0.59 to 0.66), suggesting that although these relational processes are interrelated, they remain sufficiently distinct to justify separate interpretation. Model fit indices indicated an acceptable fit to the data (χ^2^ = 42.81, *df* = 7, *p* < 0.001; RMSEA = 0.089; TLI = 0.958). While the RMSEA was slightly above the conventional threshold of 0.08, the overall fit pattern was consistent with a theoretically interpretable and psychometrically coherent multidimensional structure.

A CFA was subsequently performed to evaluate the stability of the three-factor solution identified in the EFA and to test whether a more constrained unidimensional representation could adequately account for the latent structure of Dimension C. The three-factor CFA demonstrated acceptable-to-good fit (χ^2^ = 61.34, *df* = 17, *p* < 0.001; CFI = 0.964; TLI = 0.946; RMSEA = 0.081), with standardized loadings ranging from 0.62 to 0.94, all statistically significant (*p* < 0.001).

In contrast, the unidimensional model demonstrated poor global fit (χ^2^ = 512.87, *df* = 20, *p* < 0.001; CFI = 0.812; TLI = 0.752; RMSEA = 0.192), indicating that a single latent factor is insufficient to adequately represent the complexity of the social-relational processes encompassed within this dimension.

Taken together, the EFA and CFA findings suggest that Dimension C is better conceptualized as an internally multidimensional domain composed of multiple interrelated facets of relational wellbeing, including relational closeness, social belonging, and interpersonal autonomy. Although these facets demonstrated meaningful inter-factor associations, the poor fit of the unidimensional model indicates that they should not be interpreted as a psychometrically homogeneous construct.

Overall, these findings provide preliminary support for the factorial organization of Dimension C within the Portuguese sample and reinforce the interpretation of relational wellbeing as a complex and multidimensional domain within the 7DHW framework.

#### 4.2.4 Dimension D

An EFA using the minimum residual extraction method with oblimin rotation was performed on the Dimension D items to investigate the factorial structure underlying environmental attitudes, ecological concern, and nature-based engagement. The data demonstrated high suitability for factor analysis, with a KMO value of KMO = 0.892 and a significant Bartlett's test of sphericity (χ^2^ = 3098.44, *df* = 28, *p* < 0.001).

Based on eigenvalues greater than one (2.81, 1.61, 1.24, and 1.08), a four-factor solution was retained, explaining 68.8% of the total variance (Factor 1: 28.1%, Factor 2: 16.2%, Factor 3: 12.9%, Factor 4: 11.6%). The factor structure revealed four conceptually meaningful components: (1) perceived climate change impacts, (2) environmental health threats, (3) ecosystem-based mitigation beliefs, and (4) personal engagement in nature-based practices. Factor loadings ranged from 0.49 to 0.95, and uniqueness values remained acceptable (0.19–0.52), indicating good representation of the latent constructs.

Inter-factor correlations ranged from 0.43 to 0.71, supporting the interpretation of the factors as related but distinguishable facets of environmental wellbeing. Model fit indices suggested an acceptable, although somewhat marginal, fit to the data (χ^2^ = 21.73, *df* = 2, *p* < 0.001; RMSEA = 0.112; TLI = 0.918). The elevated RMSEA should be interpreted with caution, given the small degrees of freedom and the conceptual breadth of the items, but the overall solution remained theoretically coherent and psychometrically defensible.

To assess factorial stability, a CFA was conducted based on the four-factor structure identified in the EFA. The correlated four-factor CFA model demonstrated acceptable-to-good fit (χ^2^ = 72.48, *df* = 16, *p* < 0.001; CFI = 0.956; TLI = 0.934; RMSEA = 0.086), with standardized factor loadings ranging from 0.49 to 0.95, all significant at *p* < 0.001.

In contrast, the unidimensional model demonstrated poorer fit (χ^2^ = 238.91, *df* = 20, *p* < 0.001; CFI = 0.918; TLI = 0.889; RMSEA = 0.124), indicating that a single-factor representation does not adequately capture the complexity of environmental beliefs, attitudes, and behaviors encompassed within this dimension.

Taken together, these findings suggest that Dimension D is better conceptualized as an internally multidimensional domain composed of multiple interrelated facets of environmental and ecopsychological wellbeing. The differentiation between climate-related perceptions, environmental health concerns, ecological mitigation beliefs, and personal nature engagement appears theoretically coherent and statistically distinguishable within the Portuguese sample.

Overall, the EFA and CFA results provide preliminary support for the factorial organization of Dimension D and reinforce the interpretation of environmental wellbeing as a complex and multidimensional construct within the 7DHW framework.

#### 4.2.5 Dimension E

An EFA was conducted on the Dimension E items. The minimum residual (MINRES) extraction method with oblimin rotation was used to examine the factorial structure of workplace wellbeing and environmental conditions. Sampling adequacy was good (KMO = 0.845). Bartlett's test of sphericity was significant (χ^2^ = 3, 012.67, *df* = 45, *p* < 0.001), supporting the suitability of the data for factor analysis.

Based on eigenvalues greater than one (2.21, 1.94, 1.68, and 1.12), a four-factor solution was retained, accounting for 60.6% of the total variance (Factor 1: 19.1%, Factor 2: 17.0%, Factor 3: 14.8%, Factor 4: 9.7%). The factors corresponded to four conceptually coherent domains: (1) workplace infrastructure and physical/organizational conditions, (2) meaningfulness and inclusivity of work, (3) work-life balance and financial wellbeing, and (4) physical presence and office engagement. Factor loadings ranged from 0.45 to 0.93, with acceptable uniqueness values (0.21–0.58), suggesting solid construct representation across the item set.

The inter-factor correlations ranged from 0.02 to 0.76. Some components were only weakly associated, while others showed moderate to strong overlap. This pattern supports interpreting Dimension E as partially independent but integrated. Model fit indices showed good fit (χ^2^ = 25.91, *df* = 11, *p* = 0.007; RMSEA = 0.047; TLI = 0.979), indicating that the four-factor EFA solution fits the covariance matrix well.

A CFA was then conducted to evaluate the stability of the four-factor structure identified in the EFA and to assess whether a unidimensional solution could adequately represent the latent structure of workplace wellbeing. The four-factor CFA model demonstrated good fit (χ^2^ = 84.52, *df* = 29, *p* < 0.001; CFI = 0.958; TLI = 0.939; RMSEA = 0.069), with standardized loadings ranging from 0.45 to 0.93, all statistically significant (*p* < 0.001).

In contrast, the unidimensional model demonstrated substantial misfit (χ^2^ = 801.64, *df* = 35, *p* < 0.001; CFI = 0.732; TLI = 0.661; RMSEA = 0.181), suggesting that workplace wellbeing within this framework is not adequately represented by a single homogeneous latent factor.

Taken together, the EFA and CFA findings suggest that Dimension E is better conceptualized as an internally multidimensional domain composed of multiple interrelated facets of occupational wellbeing, including organizational infrastructure, work meaning, wellbeing balance, and workplace engagement. The poor performance of the unidimensional model further indicates that occupational wellbeing experiences within the 7DHW framework are heterogeneous and multifaceted rather than psychometrically unitary.

Overall, these findings provide preliminary support for the factorial organization of Dimension E within the Portuguese sample and reinforce the interpretation of workplace wellbeing as a complex and multidimensional construct within the 7DHW framework.

#### 4.2.6 Dimension F

An EFA was conducted on the Dimension F items using the MINRES extraction method with oblimin rotation to assess the latent structure of health-related behaviors, dietary patterns, and attitudes toward psychological and medical care. Sampling adequacy was moderate (KMO = 0.648), and Bartlett's test of sphericity was significant (χ^2^ = 1, 748.23, *df* = 91, *p* < 0.001), indicating that the correlation matrix was suitable for factor analysis despite the heterogeneity of the item set.

Based on eigenvalues greater than one (2.08, 1.92, 1.63, 1.28, and 1.11), a five-factor solution was retained, accounting for 42.6% of the total variance (Factor 1: 11.4%, Factor 2: 10.6%, Factor 3: 9.1%, Factor 4: 6.3%, Factor 5: 5.2%). These factors captured five conceptually distinct domains: (1) medical care behaviors, (2) restrictive dietary practices, (3) attitudes toward psychological care, (4) food access and food processing, and (5) metabolic self-perception. Factor loadings ranged from 0.42 to 0.95, while uniqueness values were comparatively higher (0.28–0.67), reflecting the broader heterogeneity of this domain.

Inter-factor correlations were weak to moderate, ranging from −0.31 to 0.29, and included several negative associations, indicating that the latent constructs are only partially integrated. Nonetheless, model fit indices supported the adequacy of the multidimensional solution (χ^2^ = 61.92, *df* = 31, *p* = 0.001; RMSEA = 0.039; TLI = 0.948), suggesting that the five-factor structure captured the data well despite the conceptual breadth of the items.

A CFA was subsequently performed to evaluate the stability of the five-factor model identified in the EFA and to test a unidimensional alternative. The five-factor CFA demonstrated good fit (χ^2^ = 138.44, *df* = 72, *p* < 0.001; CFI = 0.942; TLI = 0.921; RMSEA = 0.048), with standardized loadings ranging from 0.42 to 0.95, all statistically significant (*p* < 0.001).

In contrast, the unidimensional model demonstrated severe misfit (χ^2^ = 1, 124.81, *df* = 77, *p* < 0.001; CFI = 0.361; TLI = 0.247; RMSEA = 0.142), indicating that the items are not adequately represented by a single homogeneous latent construct.

Taken together, these findings suggest that Dimension F is better conceptualized as an internally multidimensional domain composed of several partially independent but interrelated aspects of health behavior and self-regulation, including preventive care, dietary restriction, psychological help-seeking attitudes, food environment, and metabolic self-perception. The substantial deterioration in fit observed in the unidimensional model further reinforces the interpretation of this dimension as multifaceted rather than psychometrically unitary.

Overall, the EFA and CFA findings provide preliminary support for the factorial organization of Dimension F within the Portuguese sample. Nevertheless, given the relatively low internal consistency observed for the overall dimension, these findings should be interpreted cautiously and warrant further investigation in future psychometric studies involving independent samples and potential item refinement procedures.

#### 4.2.7 Dimension G

An EFA was conducted on the Dimension G items using the MINRES extraction method with oblimin rotation to investigate the structure of psychological flourishing in the Portuguese sample. Sampling adequacy was excellent, as indicated by a global KMO value of KMO = 0.912, and Bartlett's test of sphericity was highly significant (χ^2^ = 5, 486.73, *df* = 231, *p* < 0.001), confirming strong inter-item correlations and suitability for factor analysis.

Based on eigenvalues greater than one and inspection of the scree pattern, a six-factor solution was retained, explaining 49.0% of the total variance (Factor 1: 10.2%, Factor 2: 9.5%, Factor 3: 8.7%, Factor 4: 8.1%, Factor 5: 7.2%, Factor 6: 5.3%). The extracted factors corresponded closely to the core dimensions of the PERMA model, complemented by a distinct flow-related component: positive emotions, engagement, relationships, meaning, accomplishment, and flow. Factor loadings ranged from 0.48 to 0.88, while uniqueness values were moderate (0.26–0.64), indicating satisfactory representation of these conceptually diverse wellbeing facets.

Inter-factor correlations ranged from 0.08 to 0.56, indicating low to moderate associations between the latent constructs and supporting the interpretation of these constructs as related yet distinct components of flourishing. Model fit indices further supported the adequacy of the EFA solution (χ^2^ = 274.62, *df* = 114, *p* < 0.001; RMSEA = 0.046; TLI = 0.949), suggesting a good overall fit to the data. Although flow-related items showed somewhat greater heterogeneity, the general factor pattern remained theoretically coherent and psychometrically sound.

A confirmatory factor analysis (CFA) was subsequently conducted to evaluate the stability of the six-factor solution identified in the EFA and to assess whether a unidimensional model could adequately represent the broader construct of flourishing. The six-factor CFA demonstrated good fit (χ^2^ = 512.88, *df* = 237, *p* < 0.001; CFI = 0.952; TLI = 0.941; RMSEA = 0.053), with standardized loadings ranging from 0.48 to 0.88, all statistically significant.

In contrast, the unidimensional model demonstrated substantial misfit (χ^2^ = 1, 938.74, *df* = 252, *p* < 0.001; CFI = 0.701; TLI = 0.671; RMSEA = 0.098), indicating that flourishing within the 7DHW framework is not adequately represented by a single homogeneous latent factor. This pattern was particularly evident among the flow-related items, several of which demonstrated weaker or mixed loadings, potentially reflecting the conceptual complexity and multidimensional nature of optimal experience.

Taken together, the EFA and CFA findings suggest that Dimension G is better conceptualized as an internally multidimensional domain encompassing multiple interrelated facets of psychological flourishing. These facets include meaning, emotional wellbeing, social connectedness, engagement, self-development, and optimal experience processes. The poor performance of the unidimensional model further reinforces the interpretation of flourishing as a constellation of related but distinguishable psychological capacities rather than a psychometrically unitary construct.

Overall, these findings provide preliminary support for the factorial organization of Dimension G within the Portuguese sample and reinforce the interpretation of flourishing as a complex and multidimensional domain within the 7DHW framework. Future studies using independent samples and cross-validation procedures are nevertheless needed to further examine the stability and generalizability of these factorial structures.

### 4.3 Multidimensionality within dimensions

An important finding of the present study is that several of the proposed wellbeing dimensions demonstrated internally multifactorial structures rather than strict unidimensionality. Rather than contradicting the holistic framework of the 7DHW model, these findings may suggest that broad wellbeing domains, such as self-esteem, environmental wellbeing, flourishing, and workplace wellbeing, are composed of multiple interrelated psychological and contextual subcomponents.

This interpretation aligns with contemporary multidimensional approaches to wellbeing measurement, which increasingly conceptualize wellbeing constructs as hierarchically organized and internally heterogeneous rather than psychometrically simple latent traits. Therefore, the present findings suggest that the 7DHW dimensions may function more appropriately as higher-order wellbeing domains composed of distinguishable subfacets.

Future research should further investigate these hierarchical structures using bifactor models, second-order CFA approaches, and independent validation samples.

### 4.4 Internal consistency and item analysis

**Figure 2:**
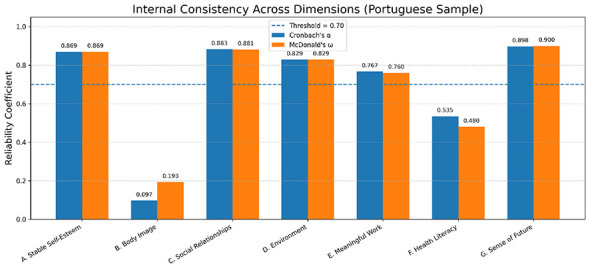
Internal consistency of the seven dimensions of the Holistic Wellbeing Questionnaire in the Portuguese sample, assessed using Cronbach's α and McDonald's ω. Dimensions A—Stable Self-Esteem, C—Social Relationships, D—Environment, and G—Sense of Future demonstrated good to excellent reliability, while Dimension E – Meaningful Work showed acceptable reliability. In contrast, Dimension B—Body Image and Dimension F—Health Literacy fell below the recommended threshold (0.70), indicating reduced internal consistency and the need for further refinement.

The internal consistency of the questionnaire was evaluated using Cronbach's α, McDonald's ω, and corrected item–total correlations (CITC) for each of the seven dimensions in the Portuguese sample. As illustrated in Figure 2, most subscales demonstrated satisfactory to excellent reliability, with α and ω values meeting or exceeding the recommended threshold of 0.70.

Specifically, strong internal consistency was observed for Dimension A—Self-Related Psychological Competencies (α = 0.869, ω = 0.869), Dimension C—Interpersonal Connectedness (α = 0.883, ω = 0.881), Dimension D—Environmental Attitudes (α = 0.829, ω = 0.829), and Dimension G—Psychological Flourishing (α = 0.898, ω = 0.900), indicating robust coherence of these constructs. The sub-scale Dimension E—Workplace Wellbeing also demonstrated acceptable internal consistency (α = 0.767, ω = 0.760), supporting its adequacy for research purposes despite its multidimensional nature.

In contrast, Dimension F—Health Behaviors and Attitudes showed lower reliability coefficients (α = 0.535, ω = 0.480), suggesting reduced internal coherence likely attributable to the conceptual breadth and heterogeneity of the items included in this dimension. Most notably, Dimension B—Body Image and Inclusivity exhibited very low internal consistency (α = 0.097, ω = 0.193), indicating that the items do not form a unified scale. This result is consistent with the factorial analyses, which demonstrated a bidimensional structure with negatively correlated factors, suggesting that this domain captures opposing constructs rather than a single latent dimension.

The CITC analysis provided further insight into the contribution of individual items to each dimension. In the reliable sub-scales (Dimensions A, C, D, E, and G), the majority of items exceeded the conventional threshold of 0.30, indicating satisfactory alignment with their respective constructs. In particular, Dimension C demonstrated consistently high corrected item–total correlations across all items, reflecting strong internal coherence of relational constructs. Similarly, Dimensions A and G showed stable and substantial CITC values, reinforcing their internal validity and conceptual consistency.

Dimension E presented a more heterogeneous pattern, with several items demonstrating strong correlations (e.g., items related to meaningful work and organizational support), while others fell closer to or below the recommended threshold. This variability suggests that although the overall scale is acceptable, certain items may benefit from refinement to improve internal coherence.

By contrast, Dimensions B and F showed consistently low CITC values across most items, confirming that their low internal consistency is not driven by isolated problematic items but reflects a broader lack of homogeneity within these constructs. In Dimension B, this pattern is likely explained by the coexistence of conceptually opposing items (e.g., body dissatisfaction vs. body acceptance), as well as potential issues related to item polarity or reverse coding. In Dimension F, the low internal consistency appears to stem from the dimension's broad conceptual scope, which integrates diverse aspects of health behavior, including medical practices, dietary habits, psychological attitudes, and perceptions of food access.

These findings suggest that both Dimension B and Dimension F would benefit from further refinement. For Dimension B, this may involve explicitly separating the construct into two sub-scales (e.g., body-related social experiences and body satisfaction) or revising item wording to improve coherence. For Dimension F, refinement could include restructuring the dimension into more clearly defined sub-domains or revising items to better align with a unified conceptual framework.

The results indicate that the majority of the Holistic Wellbeing Questionnaire's dimensions demonstrate strong psychometric properties in the Portuguese sample, with good to excellent internal consistency and coherent item contributions. Dimensions A, C, D, and G show particularly robust reliability, while Dimension E performs adequately. In contrast, Dimensions B and F exhibit substantial limitations in internal consistency, underscoring the need for targeted revision before these sub-scales can be used reliably in applied or comparative research. Nonetheless, the instrument's overall structure remains theoretically coherent and empirically supported, thereby reinforcing the validity of the multidimensional 7DHW framework in the Portuguese context.

Table 2 summarizes the internal consistency coefficients for all dimensions of the questionnaire in the Portuguese sample.

**Table 2:** Internal consistency of the seven dimensions of the Holistic Wellbeing Questionnaire in the Portuguese sample (*N* = 408).

Dimension	Construct	α	ω	Interpretation
A	Self-related psychological competencies	0.869	0.869	Good
B	Body image and inclusivity	0.097	0.193	Very poor
C	Interpersonal connectedness	0.883	0.881	Good
D	Environmental attitudes	0.829	0.829	Good
E	Workplace wellbeing	0.767	0.760	Acceptable
F	Health behaviors and attitudes	0.535	0.480	Low
G	Psychological flourishing	0.898	0.900	Excellent

### 4.5 Differentiation analysis

To examine known-groups validity and the ability of the Holistic Scale of Body and Mental Health and Wellbeing to differentiate between demographic groups in the Portuguese sample, analyses were conducted comparing participants by gender (independent-samples *t*-tests with Welch's correction) and age (one-way ANOVAs across eight categories: 18–20, 21–30, 31–40, 41–50, 51–60, 61–70, 71–80, and >80). Effect sizes for gender comparisons were estimated using Cohen's *d*, and age-related effects were interpreted based on the ANOVA results and the observed mean patterns across age groups. Because Dimensions B and F showed reduced internal consistency, any group differences involving these domains should be interpreted with caution.

#### 4.5.1 Gender differences (independent *t*-tests)

- **Dimension A—Self-Related Psychological Competencies**. Men (*n* = 122, *M* = 5.25, *SD* = 0.76) scored slightly higher than women (*n* = 286, *M* = 5.14, *SD* = 0.80), but the difference was not statistically significant, *t* = 1.31, *p* = 0.190, *d* = 0.14. The effect was small, indicating minimal gender differentiation in this domain.
- **Dimension B—Body Image and Inclusivity**. Men (*M* = 3.94, *SD* = 0.78) reported slightly higher scores than women (*M* = 3.86, *SD* = 0.70), but the difference was not significant, *t* = 1.05, *p* = 0.296, *d* = 0.12. The effect size was very small.
- **Dimension C—Interpersonal Connectedness**. Women (*M* = 5.64, *SD* = 0.95) scored somewhat higher than men (*M* = 5.51, *SD* = 0.96), but this difference was not statistically significant, *t* = −1.27, *p* = 0.205, *d* = −0.14. The effect was small.
- **Dimension D—Environmental Attitudes**. Women (*M* = 5.96, *SD* = 0.75) scored significantly higher than men (*M* = 5.71, *SD* = 0.86), *t* = −2.85, *p* = 0.005, *d* = −0.32. The effect was small to approaching moderate, suggesting greater environmental concern or ecological engagement among female participants.
- **Dimension E—Workplace Wellbeing**. Men (*M* = 5.29, *SD* = 0.79) showed slightly higher scores than women (*M* = 5.13, *SD* = 0.86), but the difference did not reach conventional significance, *t* = 1.73, *p* = 0.085, *d* = 0.18. This pattern suggests a small, non-significant tendency for men to report somewhat higher workplace wellbeing.
- **Dimension F—Health Behaviors and Attitudes**. Women (*M* = 3.69, *SD* = 0.63) scored significantly higher than men (*M* = 3.52, *SD* = 0.58), *t* = −2.63, *p* = 0.009, *d* = −0.28. The effect was small, indicating modest gender differences in health-related behaviors and attitudes.
- **Dimension G—Psychological Flourishing**. Women (*M* = 5.00, *SD* = 0.72) and men (*M* = 4.96, *SD* = 0.69) did not differ significantly, *t* = −0.52, *p* = 0.603, *d* = −0.06. The effect was negligible.

##### 4.5.1.1 Summary

Gender differences in the Portuguese sample were significant only for *Dimension D—Environmental Attitudes* and *Dimension F—Health Behaviors and Attitudes*, with women reporting higher scores in both domains. A small non-significant tendency was observed for men to report higher scores in *Dimension E—Workplace Wellbeing*. All other dimensions showed no statistically meaningful gender differences, and effect sizes were generally small. The descriptive means and dispersion patterns are consistent with the attached Portuguese reference summaries. Given the low internal consistency observed for Dimensions B and F, the corresponding group comparison results should be interpreted with caution. Although statistically significant differences emerged in some comparisons, these findings are considered exploratory and may not reflect stable latent constructs. Further psychometric refinement and replication studies are therefore needed before drawing stronger inferential conclusions regarding these dimensions.

#### 4.5.2 Age differences (ANOVAs)

One-way ANOVAs across the eight age groups revealed the following:

- **Dimension A—Self-Related Psychological Competencies**. Significant age differences were observed, *F*_(7, 400)_ = 6.03, *p* < 0.001. Scores were highest in adulthood, particularly in the 31–40 and 51–60 age groups, and declined in later life, especially among participants aged 61–80.
- **Dimension B—Body Image and Inclusivity**. Significant age differences were also found, *F*_(7, 400)_ = 3.94, *p* < 0.001. Body-related scores were relatively higher in younger adults, especially those aged 18–30, and tended to decline in mid- and later adulthood, although the small >80 group showed a slight recovery.
- **Dimension C—Interpersonal Connectedness**. Differences across age groups were significant, *F*_(7, 400)_ = 7.84, *p* < 0.001. Scores peaked in young and middle adulthood (particularly 21–60 years) and declined after age 60, suggesting reduced social connectedness in later life.
- **Dimension D—Environmental Attitudes**. Significant age-related variation was found, *F*_(7, 400)_ = 4.28, *p* < 0.001. Environmental scores remained high overall, but tended to peak in midlife and decline somewhat in older age groups, especially after age 60.
- **Dimension E—Workplace Wellbeing**. No significant age differences were observed, *F*_(7, 400)_ = 0.57, *p* = 0.780. Scores remained relatively stable across the adult lifespan.
- **Dimension F—Health Behaviors and Attitudes**. Age differences did not reach statistical significance, *F*_(7, 400)_ = 1.75, *p* = 0.097. Although some descriptive variation was present, the overall pattern suggests relative stability across age groups.
- **Dimension G—Psychological Flourishing**. Significant age differences were identified, *F*_(7, 400)_ = 5.33, *p* < 0.001. Scores were highest in younger and middle adulthood, particularly in the 31–40 age group, and declined in older participants, especially after age 60.

##### 4.5.2.1 Summary

Age-related differences were observed across several dimensions of the 7DHW framework, particularly in *Dimensions A, B, C, D*, and *G*, suggesting that self-related functioning, body image, relational wellbeing, environmental attitudes, and flourishing may vary across different stages of the lifespan in the Portuguese population. In contrast, *Dimensions E* and *F* showed comparatively greater stability across age groups. Overall, these patterns align with the descriptive Portuguese reference summaries and reinforce the interpretation of holistic wellbeing as a dynamic and multidimensional construct influenced by developmental and contextual factors. Nevertheless, some of these age-related findings should be interpreted cautiously, particularly for dimensions presenting lower internal consistency or complex multifactorial structures. Although the observed differences may reflect meaningful developmental variation, further psychometric refinement and replication using independent samples are necessary to establish the stability and generalizability of these patterns across age groups.

**Figure 3:**
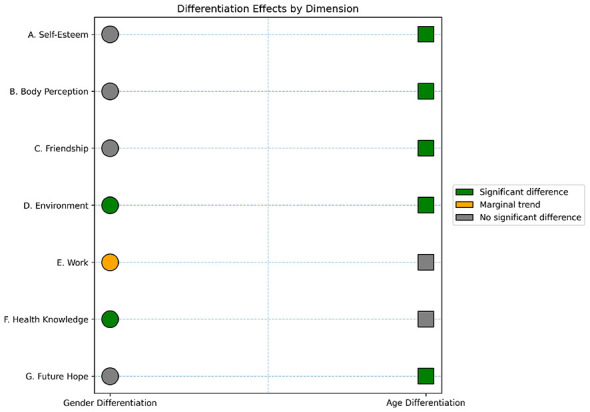
Differentiation effects across dimensions by gender (circles) and age (squares). Green indicates statistically significant differences, yellow indicates marginal trends, and gray indicates no significant differences. The order of dimensions follows the structure of the scale, from A (Self-Esteem, top) to G (Future Hope, bottom).

To visually summarize these findings, Figure 3 can be used to display the differentiation effects across dimensions by gender and age. In the Portuguese sample, significant gender effects would be visible for *Dimensions D* and *F*, while age effects would emerge for *Dimensions A, B, C, D*, and *G*. *Dimensions E* and *F* would appear relatively stable across age groups, whereas *Dimensions A, C*, and *G* would show clearer life-course variation. Because *Dimensions B* and *F* showed lower internal consistency, any differentiation involving these domains should be interpreted cautiously.

Overall, the differentiation analyses provide additional support for the scale's known-groups validity in the Portuguese sample. The observed gender differences in environmental attitudes and health-related behaviors are theoretically plausible and consistent with the descriptive reference patterns. Likewise, the age-related variation in self-related functioning, body image, social connectedness, environmental attitudes, and flourishing suggests that these dimensions are shaped by developmental stage and accumulated experience. Conversely, the relative stability of workplace wellbeing and health-related attitudes across age indicates that not all wellbeing domains are equally sensitive to demographic variation. Taken together, these findings suggest that some dimensions of the instrument may be sensitive to demographic variation in theoretically plausible ways. However, given the exploratory nature of these analyses, the generally small effect sizes, and the lower internal consistency observed in some dimensions, these results should be interpreted cautiously and viewed as preliminary rather than confirmatory evidence of group differentiation.

### 4.6 Discussion

This study aimed to evaluate the construct and known-groups validity of the Holistic Scale of Body and Mental Health and Wellbeing within a Portuguese population. Building on the factorial structure established for Dimensions A–G, the differentiation analyses provide further insight into how these domains vary across demographic groups, particularly by gender and age. Together, these findings contribute to understanding the extent to which the instrument captures both stable and context-sensitive aspects of wellbeing.

#### 4.6.1 Gender differences

The analysis of gender differences revealed a generally consistent pattern across most dimensions, with only a limited number of statistically meaningful differences. No significant gender differences were observed for *Self-Esteem* (Dimension A), although men reported slightly higher scores (men: *M* = 5.25, *SD* = 0.76; women: *M* = 5.14, *SD* = 0.80; *d* = 0.14). This pattern suggests that self-related psychological functioning is relatively stable across gender, consistent with evidence that gender differences in self-esteem tend to be small in adulthood (Orth and Robins, 2014).

Similarly, *Body Image* (Dimension B) did not show significant gender differences (men: *M* = 3.94, women: *M* = 3.86; *d* = 0.12). Although prior research often reports gender disparities in body-related perceptions (Tiggemann, 2004; Frederick and Essayli, 2016), the absence of differences in this case should be interpreted cautiously, given the dimension's low internal consistency, which may limit its measurement precision.

Significant gender differences were identified for *Environmental Attitudes* (Dimension D), with women reporting higher scores than men (women: *M* = 5.96, men: *M* = 5.71; *d* = −0.32). This finding is consistent with literature suggesting that women tend to express stronger environmental concern and engagement (Zelezny et al., 2000). A similar pattern was observed for *Health Behaviors and Attitudes* (Dimension F), where women scored higher than men (women: *M* = 3.69, men: *M* = 3.52; *d* = −0.28), reflecting gender differences in preventive health behaviors (Courtenay, 2000). However, as noted, caution is warranted in interpreting Dimension F due to its lower reliability.

No significant differences were found for *Social Relationships* (Dimension C), *Workplace Wellbeing* (Dimension E), or *Psychological Flourishing* (Dimension G), with effect sizes close to zero. These findings suggest that these domains represent more general aspects of wellbeing that are not strongly differentiated by gender.

##### 4.6.1.1 Summary

Gender differences were modest and primarily observed in environmental and health-related domains, while core psychological and social dimensions remained largely stable across gender.

#### 4.6.2 Age differences

Age-related analyses revealed more pronounced variation across several dimensions. Significant differences were observed for *Self-Esteem* (Dimension A; *F* = 6.03, *p* < 0.001), *Body Image* (Dimension B; *F* = 3.94, *p* < 0.001), *Social Relationships* (Dimension C; *F* = 7.84, *p* < 0.001), *Environmental Attitudes* (Dimension D; *F* = 4.28, *p* < 0.001), and *Sense of Future* (Dimension G; *F* = 5.33, *p* < 0.001).

Across these dimensions, a general life-course pattern emerged. Self-related competencies and psychological flourishing tended to increase from early adulthood into midlife and decline in older age groups. This trajectory is consistent with research suggesting that psychological resources and perceived wellbeing often peak during midlife and may decrease due to changes in health, social roles, and future outlook (Orth and Robins, 2014).

*Social Relationships* also showed significant variation, with higher levels observed in younger and middle-aged groups and a decline in later life. This aligns with theoretical perspectives suggesting that social networks become more selective with age (Carstensen, 1992).

*Environmental Attitudes* remained relatively high across all age groups but showed moderate variation, with slightly lower scores among older participants. This may reflect generational differences in environmental engagement or in perceived agency to address ecological challenges (Dietz, 2007).

No significant age-related differences were found for *Workplace Wellbeing* (Dimension E; *p* = 0.780) or *Health Behaviors and Attitudes* (Dimension F; *p* = 0.097), suggesting that these domains may be less sensitive to age-related variation.

##### 4.6.2.1 Summary

Age differences were evident across multiple dimensions, particularly those related to self-perception, social functioning, and future orientation, highlighting the role of developmental processes in shaping wellbeing.

#### 4.6.3 Implications for known-groups validity

The differentiation patterns observed in this study provide preliminary and exploratory evidence that some dimensions of the instrument may differentiate between demographic groups in theoretically plausible ways. The instrument demonstrated sensitivity to group differences in domains where theoretical expectations of variation are greatest, particularly in environmental attitudes, health-related behaviors, and age-dependent changes in psychological and social functioning.

Significant gender differences were identified in *Environmental Attitudes* (Dimension D; *d* = −0.32) and *Health Behaviors and Attitudes* (Dimension F; *d* = −0.28), with women reporting higher scores in both domains. These findings are consistent with the existing literature, which indicates that women tend to exhibit greater engagement in pro-environmental behavior and preventive health practices, often linked to socialization processes emphasizing care, responsibility, and risk awareness (Zelezny et al., 2000; Courtenay, 2000). These patterns may indicate sensitivity to behavioral and attitudinal variation, although replication in independent samples is needed.

Age-related differentiation further strengthens the evidence for known-groups validity. Several dimensions, including *Self-Esteem* (Dimension A), *Social Relationships* (Dimension C), and *Sense of Future* (Dimension G), exhibited systematic variation across age groups. The observed inverted-U trajectory, with higher levels in early and middle adulthood followed by declines in later life, aligns with well-documented patterns in lifespan psychology and wellbeing research (Orth and Robins, 2014). Similarly, the variation observed in social relationships is consistent with socioemotional selectivity theory, which posits that social networks become more selective and functionally focused with age (Carstensen, 1992).

The scale also captured variation in *Environmental Attitudes* across age groups (Dimension D), supporting the idea that ecological awareness is shaped by generational experiences and exposure to environmental discourse (Dietz, 2007). These results suggest that the instrument can reflect both developmental and contextual influences on wellbeing, further reinforcing its predictive capacity.

At the same time, the absence of significant differences in certain domains, such as *Workplace Wellbeing* (Dimension E) across both gender and age, and *Psychological Flourishing* (Dimension G) across gender, indicates that the instrument also captures core aspects of wellbeing that remain relatively stable across demographic groups. This balance between sensitivity to expected variation and stability in more universal constructs is a key characteristic of a robust measurement tool. It suggests that the scale is not merely detecting superficial differences but rather reflects meaningful distinctions that are theoretically justified while maintaining consistency across the foundational dimensions of wellbeing.

Importantly, the instrument's multidimensional structure contributes to this balance. Contemporary models of wellbeing emphasize that psychological health comprises multiple interrelated yet distinct components, including emotional, social, behavioral, and existential dimensions (Ryff, 1989; Seligman, 2011). The present findings support this perspective by demonstrating that different dimensions respond differently to demographic factors, highlighting the importance of assessing wellbeing as a constellation of related yet independent constructs.

Nevertheless, interpreting differentiation patterns in *Body Image* (Dimension B) and *Health Behaviors and Attitudes* (Dimension F) should be approached with caution, given their lower internal consistency. While some expected patterns were observed, measurement limitations may have attenuated or obscured true effects. This underscores the importance of ensuring psychometric robustness when evaluating known-groups validity.

Taken together, the results indicate that the instrument possesses strong known-groups validity, as it successfully captures variation across key domains of wellbeing in ways that are theoretically coherent and empirically grounded. At the same time, the stability observed in other dimensions reinforces its capacity to measure enduring aspects of human functioning. This combination of sensitivity and robustness supports the use of the scale as a comprehensive framework for assessing holistic wellbeing across diverse populations and contexts.

#### 4.6.4 Limitations and future directions

Several limitations should be acknowledged. First, the distribution of participants across age groups was uneven, particularly in older age categories, which may limit the generalizability of age-related findings. Future studies should aim for more balanced sampling across the lifespan.

Second, the low internal consistency observed for *Dimension B* (Body Image; α = 0.097, ω = 0.193) and *Dimension F* (Health Behaviors and Attitudes; α = 0.535, ω = 0.480) represents a significant limitation. These results indicate that the items within these dimensions do not form coherent constructs, which may have influenced both the factorial and differentiation analyses. Although exploratory group comparisons were retained for descriptive and hypothesis-generating purposes, these findings should be interpreted cautiously, as reduced reliability may compromise the stability and interpretability of the observed effects. Future studies should therefore prioritize item refinement, dimensional restructuring, and independent validation procedures to strengthen the psychometric robustness of these domains.

Another important limitation concerns the use of the same sample for both exploratory and confirmatory factor analyses. Although this approach is frequently adopted in preliminary psychometric studies with moderate sample sizes, it may inflate model fit indices and limit the generalizability of the factorial solutions obtained. Future studies should therefore replicate the factorial structure using independent validation samples and cross-validation procedures.

In addition, an a priori power analysis was not conducted before data collection, which should be considered a limitation of the present study. Nevertheless, the final sample size (*N* = 408) exceeded commonly recommended minimum thresholds for exploratory and confirmatory factor analyses in psychometric research.

In addition, further validation across diverse populations is needed to ensure the instrument's robustness and applicability. Understanding how wellbeing dimensions are expressed across different contexts will be essential for strengthening both theoretical and practical contributions.

Furthermore, future studies should also consider including socioeconomic positioning, including subjective socioeconomic status, and sociopolitical orientation variables, as previous literature suggests that these factors may influence perceptions of wellbeing, environmental attitudes, and health-related behaviors (Operario et al., 2004; Fors Connolly and Lindh, 2025; Wang et al., 2023).

In conclusion, the findings provide preliminary support for aspects of the factorial structure and known-groups differentiation patterns observed in this sample. While some dimensions require refinement, the instrument demonstrates solid potential as a multidimensional framework for assessing wellbeing.

## 5 Conclusion

The present study provides an initial psychometric evaluation of the Portuguese version of the Holistic Scale of Body and Mental Health and Wellbeing. The findings provide preliminary support for several psychometric properties as a multidimensional assessment tool. The results support the conceptual basis of the 7DHW model, demonstrating that wellbeing is best conceptualized as a constellation of interrelated psychological, social, environmental, and behavioral domains.

The factorial analyses suggested that all seven dimensions reflect structured, interpretable latent constructs, strengthening the multidimensional nature of holistic wellbeing. Reliability findings further indicated that most dimensions, particularly Self-Esteem, Social Relationships, Environment, and Sense of Future, show strong internal consistency, supporting their use in both research and real-world contexts. At the same time, the lower reliability observed in Body Image and Health Knowledge highlights important areas for future refinement, implying the need for improved conceptual alignment and item development.

The differentiation analyses provide additional support for the instrument's known-groups validity. The scale clearly identified meaningful differences across gender and age, particularly in environmental attitudes, health-related behaviors, and life-course changes in psychological and social functioning. These patterns indicate that the instrument is sensitive to theoretically expected differences while maintaining stability across more universal dimensions of wellbeing, such as workplace experience and overall flourishing. This balance between sensitivity and robustness is a key strength of the measure.

Importantly, the findings reaffirm the relevance of adopting holistic models for understanding wellbeing in contemporary societies. In the Portuguese context, where mental health challenges, demographic aging, and social inequalities intersect, the availability of a culturally adapted and multidimensional instrument is particularly valuable. The 7DHW questionnaire is a useful tool for clinical, organizational, educational, and public health settings, enabling more comprehensive assessment and targeted intervention.

Future research should focus on refining dimensions with lower internal consistency, exploring longitudinal trajectories of wellbeing, and validating the instrument across more diverse populations. Such efforts will further strengthen the scale's applicability and advance coordinated approaches to health and wellbeing.

In conclusion, the Portuguese version of the 7DHW questionnaire represents a theoretically based, empirically supported, and practically relevant instrument for capturing the complexity of human being wellbeing, connecting the gap between multidimensional theory and everyday assessment.

## Data Availability

The datasets presented in this article are not readily available because the dataset contains anonymized participant responses; however, it is not publicly available due to data protection and confidentiality considerations. Although no personally identifiable information was collected, the data originate from a defined participant pool and are subject to institutional and GDPR regulations. Access to the dataset may be granted by the corresponding author upon reasonable request, provided that the intended use complies with ethical standards and data protection requirements. Requests to access the datasets should be directed to Inês Santos Silva, ines.santossilva@2256c.health.
